# Use of topical methylene blue to image nuclear morphometry with a low-cost scanning darkfield microendoscope

**DOI:** 10.1117/1.JBO.29.5.050501

**Published:** 2024-05-21

**Authors:** Huayu Hou, Jennifer Carns, Richard A. Schwarz, Ann M. Gillenwater, Sharmila Anandasabapathy, Rebecca R. Richards-Kortum

**Affiliations:** a Rice University, Department of Bioengineering, Houston, Texas, United States; b The University of Texas M.D. Anderson Cancer Center, Department of Head and Neck Surgery, Houston, Texas, United States; c Baylor College of Medicine, Department of Medicine, Houston, Texas, United States

**Keywords:** fiber-optic microendoscopy, methylene blue, scanning darkfield, cancer detection

## Abstract

**Significance:**

Fiber-optic microendoscopy is a promising approach to noninvasively visualize epithelial nuclear morphometry for early cancer and precancer detection. However, the broader clinical application of this approach is limited by a lack of topical contrast agents available for *in vivo* use.

**Aim:**

The aim of this study was to evaluate the ability to image nuclear morphometry *in vivo* with a novel fiber-optic microendoscope used together with topical application of methylene blue (MB), a dye with FDA approval for use in chromoendoscopy in the gastrointestinal tract.

**Approach:**

The low-cost, high-resolution microendoscope implements scanning darkfield imaging without complex optomechanical components by leveraging programmable illumination and the rolling shutter of the image sensor. We validate the integration of our system and MB staining for visualizing epithelial cell nuclei by performing *ex vivo* imaging on fresh animal specimens and *in vivo* imaging on healthy volunteers.

**Results:**

The results indicate that scanning darkfield imaging significantly reduces specular reflection and resolves epithelial nuclei with enhanced image contrast and spatial resolution compared to non-scanning widefield imaging. The image quality of darkfield images with MB staining is comparable to that of fluorescence images with proflavine staining.

**Conclusions:**

Our approach enables real-time microscopic evaluation of nuclear patterns and has the potential to be a powerful noninvasive tool for early cancer detection.

## 1 Introduction

Changes in nuclear morphology, including pleomorphism and increased nuclear-to-cytoplasmic ratio, are highly predictive biomarkers for early recognition of epithelial precancerous lesions.^1^ Sub-cellular resolution fiber-optic microendoscopy, in combination with vital nuclear stains, has shown promise to improve early cancer detection by noninvasively visualizing nuclear morphometry. During *in vivo* fiber-optic microendoscopy, a thin, flexible coherent fiber bundle is placed in direct contact with the tissue epithelium to relay optical signal from the tissue surface to an external imaging system. Microscopic images with histological information, which are not readily available at the point-of-care using traditional imaging methods, can be provided to clinicians in real time for identification and delineation of precancerous lesions. Existing clinical studies have demonstrated that fiber-optic microendoscopy can effectively improve the performance of detecting precancer and early cancer in various anatomic sites, including oral cavity,^2^^,^^3^ lung,^4^ pancreas,^5^ uterine cervix,^6^^,^^7^ and gastrointestinal (GI) tract.^8^^–^^11^

To visualize nuclear morphology, exogenous contrast agents are needed to selectively stain cell nuclei. For example, proflavine is a fluorescent contrast agent that is commonly used to stain epithelial cell nuclei when imaging with fluorescence microendoscopy systems.^2^^,^^6^^,^^7^^,^^11^ Immediately after topical application of proflavine, nuclear morphology can be readily resolved with high signal-to-noise ratio. However, the use of proflavine on human subjects is often limited to investigational purposes,^12^ which limits broader clinical application of microendoscopy imaging for early cancer detection. There is a need for more readily available contrast agents to support the clinical translation of high-resolution optical imaging of nuclear morphometry.

Methylene blue (MB) is a cationic absorbing dye that binds negatively charged nucleic acid via electrostatic bonds and is an alternative topical stain for cell nuclei.^13^ MB is FDA-approved for submucosal injection for endoscopic resection in GI tract.^14^^,^^15^ The topical application of MB for chromoendoscopy is recommended in clinical guidelines for evaluation of the stomach and colon (inflammatory bowel disease) and routinely performed for detection of dysplastic lesions in GI tract.^16^^,^^17^ In addition, MB is FDA-approved for intravenous administration to treat methemoglobinemia.^13^ Numerous clinical studies have demonstrated *in vivo* application of MB on human subjects for macroscopic examination of oral precancerous lesions,^18^^,^^19^ endocytoscopy,^20^^,^^21^ and surgical guidance.^22^ MB has been verified in these studies as a safe contrast agent with significant potential for broader acceptance in clinical uses. Similar to the staining procedure with proflavine, MB can be topically applied for immediate nuclear staining. Nuclear morphology can be visualized via reflectance imaging leveraging the strong optical absorption of MB in the visible spectrum. However, a major challenge of implementing reflectance imaging through a fiber bundle is that strong internal reflection is generated at the probe surfaces, significantly reducing image quality. This limitation severely hinders the application of fiber-optic microendoscopy with MB staining to image nuclear morphometry.

We recently developed a low-cost, high-resolution fiber-optic microendoscope equipped with scanning darkfield reflectance imaging (DF-HRME).^23^ Leveraging digital light projection technology and the rolling shutter of the image sensor, scanning darkfield imaging is achieved without expensive optomechanical components, significantly suppressing the internal reflection in the fiber and enhancing image contrast. A previous study demonstrated the use of DF-HRME in characterizing angiogenesis without exogenous contrast agents for early cancer detection.^23^ In this study, we extend the capability of DF-HRME to achieve high-resolution imaging of epithelial cell nuclei with MB staining. We first evaluate the performance of scanning darkfield reflectance imaging in resolving the morphology of MB-stained cell nuclei when imaging freshly resected porcine tongue. We further validate the ability of DF-HRME to image MB-stained nuclei in the oral cavity of healthy volunteers. Our results show that DF-HRME imaging in combination with MB staining can characterize nuclear morphometry with high image contrast and resolution, providing a promising approach to improve the detection of precancer and early cancer.

## 2 Methods

### 2.1 DF-HRME System Design

**Fig. 1:**
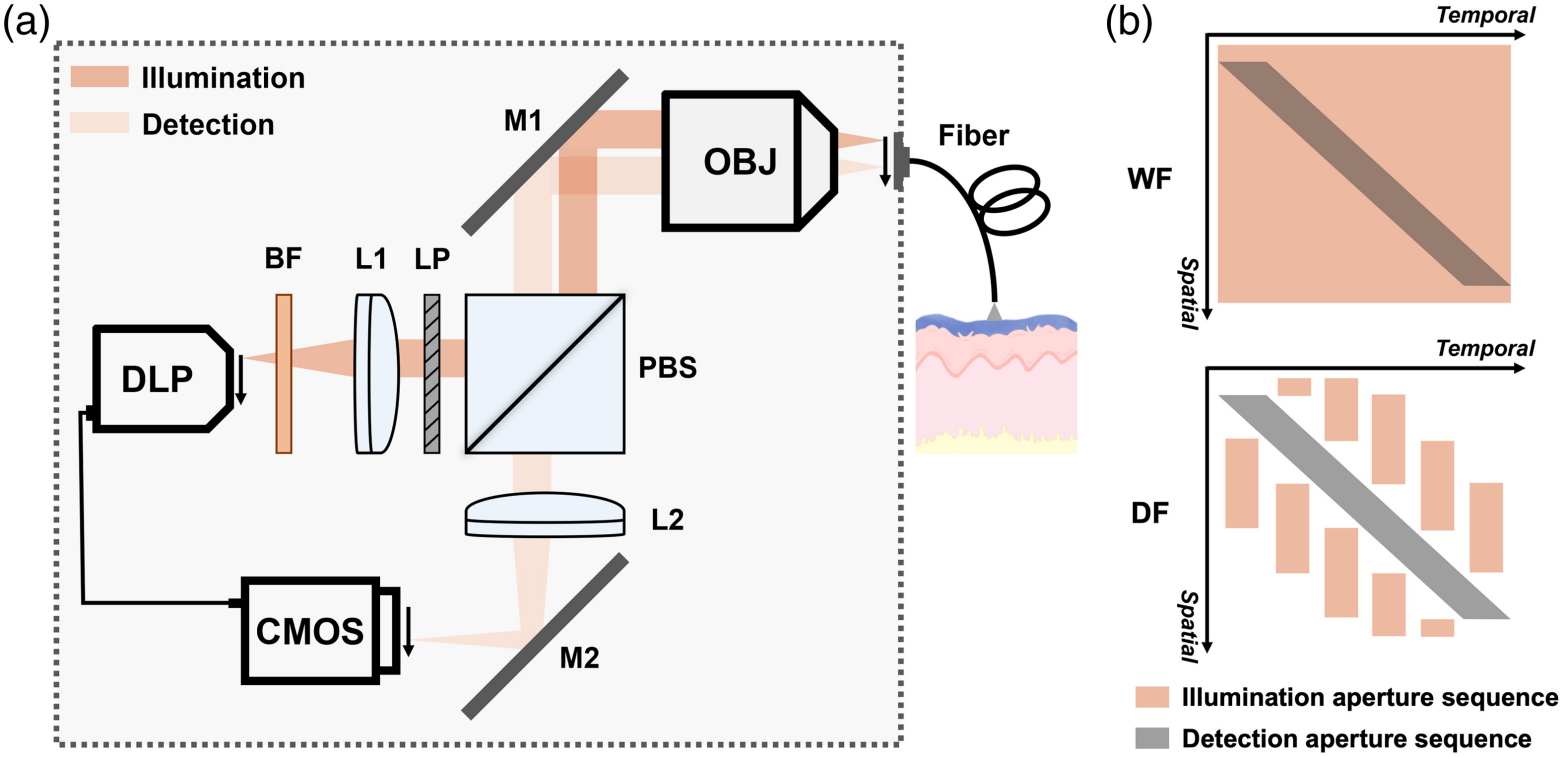
DF-HRME implements scanning darkfield imaging to reduce internal reflection and enhance image contrast. (a) Optical schematic of DF-HRME. To enable scanning darkfield imaging, a spatiotemporal offset is introduced between illumination and detection by programming and synchronizing the DLP illumination and CMOS camera rolling shutter. Arrows show the scanning direction. A thin, flexible fiber bundle is placed in contact with tissue epithelium stained by MB for real-time imaging of cell nuclei. The dashed square indicates the optical system enclosure. (b) Spatiotemporal design of illumination and detection aperture sequences. Overlapped illumination and detection aperture in non-scanning widefield imaging captures the strong specular reflection generated at fiber surfaces, degrading image quality. In scanning darkfield imaging, a sequence of illumination line pair patterns is designed to avoid overlap with the detection aperture and suppress specular reflection background. DF-HRME, scanning darkfield high-resolution microendoscope; DLP, digital light projector; CMOS, complementary metal-oxide semiconductor camera; BF, bandpass filter; LP, linear polarizer; L1, collimating lens; L2, tube lens; PBS, polarizing beamsplitter; M1, M2, mirrors; OBJ, microscope objective; WF, non-scanning widefield imaging; DF, scanning darkfield imaging.

The optical design of DF-HRME is shown in Fig. 1(a). To achieve scanning darkfield imaging without complex optomechanical components, we utilize structured illumination provided by a digital light projector (DLP) (LightCrafter 4500, Texas Instruments, Dallas, Texas) and digital line-scanning detection enabled by the rolling shutter of a complementary metal-oxide semiconductor (CMOS) image sensor (FL3-U3-120S3C-C, Teledyne FLIR, Wilsonville, Oregon). The spatiotemporal design of illumination and detection aperture sequences is shown in Fig. 1(b). For non-scanning widefield imaging, no illumination pattern is used and the entire field of view (FOV) is illuminated. The detection aperture collects the specular reflection generated at fiber surfaces, which significantly reduces contrast and image quality. When performing scanning darkfield imaging, a series of line pairs with different spatial locations are programmed as illumination patterns and projected sequentially from the DLP. A spatiotemperal offset between illumination and detection (rolling shutter) is ensured by adjusting and synchronizing the aperture sequence. Compared to non-scanning widefield imaging, scanning darkfield imaging avoids the spatial overlap between illumination and detection. Direct specular reflection is effectively rejected and only scattered signals are captured, which substantially improves contrast and the visibility of tissue features.

In the previous study, the green LED of the DLP and a green light bandpass filter centered at 525 nm were used in DF-HRME for label-free imaging of microvasculature. Hemoglobin absorption provided the endogenous contrast for microvessels.^23^ To extend the capability of DF-HRME for imaging cell nuclei with MB staining, we replaced the green light filter with a bandpass filter centered at 605 nm (605/64 nm, FF01-605/64-25, AVR Optics) and enabled both green and red LEDs of the DLP for illumination. The wavelength is selected to match the absorption peak of MB and enhance nuclear contrast. DF-HRME achieves a seamless transition between these two imaging applications via a simple filter change and software control. Through a collimating lens (AC254-100-A, Thorlabs) and a 10× objective (#86-818, Edmund Optics), projected illumination patterns are focused onto the proximal end of a thin and flexible fiber-optic imaging probe (790  μm circular FOV; FIGH-30-850N, Myriad Fiber Imaging). The fiber probe, which is attached externally to the DF-HRME, relays the structured illumination onto the tissue epithelium stained by MB and collects scattered darkfield signals. Real-time darkfield images of tissue are focused on the image sensor through the objective and a tube lens (AC254-100-A, Thorlabs). We used a linear polarizer (LPVISE100-A, Thorlabs) and a polarizing beamsplitter (CCM1-PBS251, Thorlabs), as well as background subtraction, to further suppress residual background signal. A graphical user interface (GUI) is programmed for software control and real-time data are displayed at a video frame rate (seven frames per second). The DF-HRME was assembled into a portable encasement (dashed square) to facilitate application in different clinical settings and the total cost of goods is less than $5500.

### 2.2 *Ex Vivo* Imaging of Animal Specimens

We performed *ex vivo* imaging on fresh porcine tongue specimens obtained from a local abattoir to evaluate the performance of DF-HRME imaging with MB staining for characterization of nuclear morphology. We compared images obtained in three modes: scanning darkfield reflectance imaging with MB staining, non-scanning widefield reflectance imaging with MB staining, and non-scanning widefield fluorescence imaging with proflavine staining. For a direct comparison of all three modalities, the distal end of fiber probe was controlled using a translation stage so that the same FOV was imaged with each modality. We first topically applied proflavine solution (0.01% w/v in phosphate-buffered saline) to the specimen using a cotton-tipped applicator and performed imaging using a fluorescence microendoscopy system that has been previously described.^24^ The distal end of fiber probe was affixed to a translation stage, allowing it to be translated away from the tissue surface in the vertical direction with a fixed lateral position after fluorescence imaging. This created space to wash out proflavine and stain the same tissue area with MB solution (0.3% w/v in deionized water, Biopharm). We performed DF-HRME imaging immediately after the topical administration of MB solution. We placed the distal end of the fiber in contact with MB-stained tissue epithelium and connected the proximal end of fiber to the DF-HRME for reflectance imaging. When imaging with DF-HRME, we programmed the system to record successive image frames from the same FOV using scanning darkfield imaging and non-scanning widefield imaging.

Microendoscopy images were processed to improve the visibility of nuclear morphology; we first applied a Gaussian filter to remove the intrinsic honeycomb pattern of the fiber bundle in microendoscopy images and then linearly adjusted the brightness of the images to a consistent level. Image contrast was enhanced using adaptive histogram equalization. The same image processing procedure was applied to all fluorescence, widefield reflectance, and darkfield reflectance images.

### 2.3 *In Vivo* Imaging of Healthy Volunteers

To further demonstrate the potential of DF-HRME imaging in combination with MB staining for *in vivo* microscopic evaluation of nuclear features, we performed scanning darkfield imaging in the oral cavity of healthy adult volunteers at Rice University. The study was approved by the Institutional Review Board of Rice University. Prior to each imaging experiment, written informed consent was obtained from the participant, and the imaging probe underwent standard high-level disinfection procedure. Tissue areas of interest were stained by 0.3% (w/v) MB solution using cotton-tipped applicators. We performed DF-HRME imaging immediately after the topical administration of MB solution. The fiber probe was placed in gentle contact with different stained tissue sites in the oral cavity. Microscopic images of epithelial cell nuclei were displayed on the GUI in real time and saved for further evaluation. DF-HRME imaging was conducted for 10 to 15 min to examine tissue areas of interest. The quality of MB staining and DF-HRME images was consistent during the imaging experiment. Obtained *in vivo* images were processed using the same image processing procedure described in Sec. 2.2 to improve the image quality.

## 3 Results

### 3.1 *Ex Vivo* Imaging of Animal Specimens

**Fig. 2:**
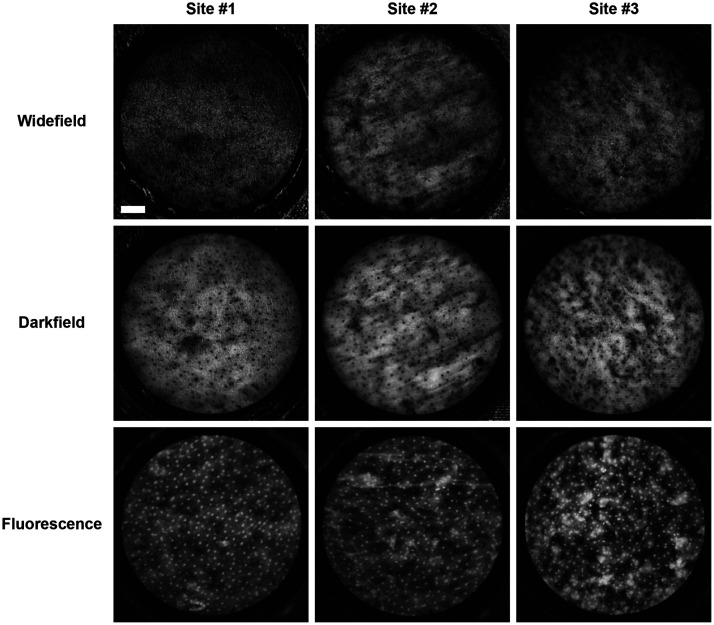
Comparison of representative images acquired from porcine tongue specimens using non-scanning widefield imaging, scanning darkfield imaging, and fluorescence imaging. The image contrast of MB stained cell nuclei is significantly improved by darkfield imaging compared to widefield imaging. Cell nuclei are clearly resolved with comparable image quality in darkfield images and corresponding fluorescence images. (Scale bar: 100  μm)

Figure 2 shows images acquired at representative sites of porcine tongue specimens using non-scanning widefield reflectance imaging and scanning darkfield reflectance imaging when staining with MB, as well as fluorescence imaging with proflavine staining. Due to optical absorption, cell nuclei stained with MB appear as dark dots when using reflectance imaging. Strong reflection background is observed in widefield images, which compromises the visibility of stained cell nuclei. Only a few cell nuclei are visualized, and it is difficult to discern morphology. In comparison, scanning darkfield imaging consistently improves image contrast and signal-to-noise ratio by effectively suppressing specular reflection. Darkfield images captured at the same tissue sites reveal the size, shape, and distribution of epithelial cell nuclei with high spatial resolution throughout the entire FOV.

As shown in Fig. 2, darkfield imaging with MB staining has comparable ability to image nuclear morphology as compared to fluorescence imaging with proflavine staining. The appearance of cell nuclei in darkfield images differs slightly from that in the corresponding fluorescence images of the same sites. The observed difference can be attributed to the rotation of the fiber proximal end and the intermediate tissue processing steps when imaging the same tissue region with these two imaging modalities. Nevertheless, similar nuclear patterns can be discerned with high spatial resolution and image contrast by both imaging modalities. We calculated the number of nuclei in 25 regions of interest with identical size from all collected image data. On average, darkfield images reveal similar nuclear count (91.4±11.4) as fluorescence images (92.0±11.0). These results demonstrate that DF-HRME imaging with MB staining is an effective approach for real-time microscopic assessment of nuclear morphometry.

### 3.2 *In Vivo* Imaging of Healthy Volunteers

**Fig. 3:**
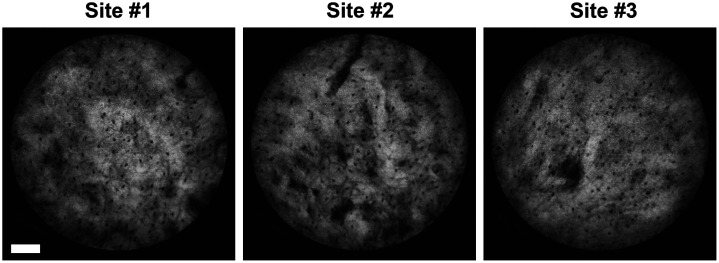
Representative DF-HRME images acquired in the oral cavity of healthy volunteers. The morphology of cell nuclei stained by MB is clearly resolved by *in vivo* DF-HRME imaging. (Scale bar: 100  μm)

Figure 3 shows representative DF-HRME images obtained from various MB-stained sites within the oral cavity of healthy normal volunteers. Stained cell nuclei with uniform size and shape are readily distinguishable in darkfield images. Each individual cell nucleus is well-isolated, and nuclei are regularly spaced in all *in vivo* images. These results indicate that DF-HRME imaging in combination with MB staining can reveal epithelial nuclear features with high spatial resolution and high contrast on healthy volunteers. This approach has the potential to enable a comprehensive evaluation of nuclear atypia associated with malignant progression in suspicious lesions, such as increased nuclear size, increased nuclear density, and nuclear pleomorphism.

## 4 Discussion and Conclusion

In this work, we demonstrate a fiber-optic microendoscopy system integrated with MB topical staining for *in vivo* imaging of epithelial nuclear morphometry in real time. DF-HRME implements scanning darkfield reflectance imaging via digital scanning, which significantly improves image contrast compared to non-scanning widefield imaging while maintaining a low system complexity and cost. *Ex vivo* imaging of porcine tongue specimens validates that the approach can resolve nuclear patterns with image quality comparable to fluorescence imaging of proflavine-stained cell nuclei. We further evaluated the *in vivo* imaging capability of DF-HRME in healthy volunteers. Our results suggest that DF-HRME imaging in combination with MB staining can be an alternative solution to noninvasively image cell nuclear morphometry for precancer and early cancer detection in real time.

The broad clinical application of existing fiber-optic microendoscopy systems is hindered by the lack of clinically approved fluorescent contrast agents. MB is a convenient and effective topical nuclear stain, which is already used for a number of clinical applications. DF-HRME overcomes the barriers of reflectance imaging of MB-stained cell nuclei caused by specular reflection in a fiber-optic system and can be readily adapted to visualize both biomarkers of precancer—nuclear atypia and dysregulated angiogenesis^23^—at a microscopic level. DF-HRME is a low-cost technology that can be easily used in various clinical settings for point-of-care diagnosis. Future studies are warranted to evaluate the clinical performance of the DF-HRME, including the effectiveness of combining microscopic information from two biomarkers to improve early cancer detection. Image analysis algorithms can be developed to automatically quantify cellular features in DF-HRME images, such as nuclear-to-cytoplasm ratio, to discriminate precancer and early cancer from benign tissue sites. If validated in future research, DF-HRME can be a powerful noninvasive tool for real-time diagnosis of neoplastic lesions without a biopsy and improve global cancer prevention.

In conclusion, we demonstrate implementation of a low-cost, portable, and easy-to-use microendoscope in combination with MB staining for real-time imaging of nuclear morphometry. Preliminary results validate that DF-HRME can effectively resolve nuclear patterns at a microscopic level with image quality comparable to fluorescence imaging. Future work can focus on further evaluating the clinical performance of DF-HRME in the detection of precancer and early cancer at the point-of-care.

## Data Availability

The data that support the findings of this article are not publicly available and can be obtained from the authors upon reasonable request.
